# Interfacial Design of Mixed Matrix Membranes via Grafting PVA on UiO-66-NH_2_ to Enhance the Gas Separation Performance

**DOI:** 10.3390/membranes11060419

**Published:** 2021-05-31

**Authors:** Saeed Ashtiani, Mehdi Khoshnamvand, Chhabilal Regmi, Karel Friess

**Affiliations:** 1 Department of Physical Chemistry, University of Chemistry and Technology, Prague, Technická 5, 16628 Prague 6, Czech Republic; regmic@vscht.cz; 2 State Key Laboratory of Environmental Chemistry and Ecotoxicology, Research Center for Eco-Environmental Sciences, Chinese Academy of Sciences, Beijing 100085, China; mehdi.khoshnam@yahoo.com

**Keywords:** metal–organic framework, mixed-matrix membrane, interface engineering, UiO-66-NH_2_

## Abstract

In this study, defect-free facilitated transport mixed matrix membrane (MMM) with high loading amount of UiO-66-NH_2_ nanoparticles as metal–organic frameworks (MOFs) was fabricated. The MOFs were covalently bonded with poly (vinyl alcohol) (PVA) to incorporate into a poly (vinyl amine) (PVAm) matrix solution. A uniform UiO-66-NH_2_ dispersion up to 55 wt.% was observed without precipitation and agglomeration after one month. This can be attributed to the high covalent interaction at interfaces of UiO-66-NH_2_ and PVAm, which was provided by PVA as a functionalized organic linker. The CO_2_ permeability and CO_2_/N_2_ and selectivity were significantly enhanced for the fabricated MMM by using optimal fabrication parameters. This improvement in gas performance is due to the strong impact of solubility and decreasing diffusion in obtained dense membrane to promote CO_2_ transport with a bicarbonate reversible reaction. Therefore, the highest amount of amine functional groups of PVAm among all polymers, plus the abundant amount of amines from UiO-66-NH_2_, facilitated the preferential CO_2_ permeation through the bicarbonate reversible reaction between CO_2_ and –NH_2_ in humidified conditions. XRD and FTIR were employed to study the MMM chemical structure and polymers–MOF particle interactions. Cross-sectional and surface morphology of the MMM was observed by SEM-EDX and 3D optical profilometer to detect the dispersion of MOFs into the polymer matrix and explore their interfacial morphology. This approach can be extended for a variety of polymer–filler interfacial designs for gas separation applications.

## 1. Introduction

It is generally accepted that CO_2_ as a gas is known as the primary source of climate change and is required to be separated from flue gas to eliminate the greenhouse effect [1]. Thus far, various types of separation techniques such as adsorption, absorption [2], cryogenic distillation [3], microbial and algal have been applied for the protection of the global environment by CO_2_ emission [4]. Although these conventional methods have been successfully employed for the separation of CO_2_, some principal drawbacks such as the immense amount of adsorbents, low adsorbing surface area, high operational and maintenance cost, and equipment corrosion problems highlight the absence of effective worldwide technology for CO_2_ separation [5,6]. Membrane-based separation processes offer a promising alternative for this purpose due to their compact design, cost-effectiveness, high surface area, ease of maintenance, and environmentally friendly nature [7]. Nevertheless, inorganic membranes possess high chemical and thermal stability with high selectivity. They are oxidized because of their low permeability due to the requirement of a thicker layer to prevent pinholes and cracks [8]. Polymeric membranes exhibit good permeability and easy processability but their low mechanical and thermal stability limit their wide range of industrial applications. Therefore, organic and inorganic hybrid membranes take advantage of both polymeric and inorganic membranes to surpass the 2008 Robeson upper bound limit where the selectivity is plotted against the CO_2_ permeability [9]. Generally, the Robeson plots and corresponding upper bounds are the benchmarks used to evaluate the membrane separation performance for various gas pairs. This type of plot can also judge the relative potential of new membrane materials for exceeding the upper bound line [10].

The most important challenges in the hybrid membrane are the strong interface interaction between the organic and the inorganic phases, and the uniform dispersion of the inorganic particles. Mixed matrix membranes (MMMs) represent a specific type of hybrid membrane forms with incorporated filler into the continuous polymer matrix. The embedded filler particles are commonly different inorganic materials with inherent superior separation characteristics such as nanoparticles, carbon molecular sieves, zeolites, etc. [11]. A metal–organic framework (MOF) is a crystalline-structured porous material, which is synthesized by the uniform arrangement of positively charged metal ions that interact with the organic linker or cluster molecules. The larger surface area, void volumes, pore connectivity, and chemical and thermal stability of the MOFs have drawn intense efforts compared to the other filler materials, especially for gas sorption and vapors [12]. As aforementioned, particle dispersion problems such as aggregation, sedimentation, and polymer–MOF interface morphology challenges such as weak adhesion between polymer and MOF, polymer chain rigidification, and MOFs pore blockage by the polymer chains, result in deterioration of gas separation performance of the MMM [13]. Therefore, obtaining a homogenous dispersion of MOFs into the polymer matrix to achieve a defect-free polymer–MOF interface and appropriate combination of polymer and MOFs are the key elements controlling the final separation performances of the MMM, which need to be addressed. Wu et al. [14], reported a dual-interfacial approach to engineering the MOF-74 to obtain a polycrystalline MOF-74 intermediate layer on the core MOF surface to divide the single MOF interface to the MOF–MOF and MOF–polymer interfaces. It resulted in the growth of the Ni-MOF-7 shell layer to prevent the gas molecules’ transport horizontally, causing increment of C_2_H_4_/C_2_H_6_ selectivity. Moreover, to obtain effective separation performances in MMM, a good MOF configuration to maximize the polymer–MOF interaction is required, forcing the gas molecules to pass through the interconnected MOF channel and the polymer matrix. Recently, Hossain et al. [15] prepared cross-linked MMM by covalently anchoring the attaching polyethylene glycol (PEG)/polypropylene glycol (PPG)–polydimethylsiloxane (PDMS) to UiO-66 using ring-opening metathesis polymerization to improve the compatibility of MOF with the polymer matrix and prevent the MOF sedimentation during the casting process. Compared to other methods, it is particularly challenging to obtain the favorable MOF–polymer interface structure using the random blending method, as long as H-bonding or *π−π* stacking is taking place instead of strong chemical bonds at the MOF–polymer interfaces. Furthermore, the mentioned methods, such as dual-interfacial approach [14], in situ polymerization [16], grafting-to, and grafting-from strategies [17], demand several complicated modification steps, high fabrication cost, and are limited by facilities, as they can be applied in lab-scale processes from a fundamental point of view. 

Recently, our group developed a simple and effective approach using the “bridging technique” [18] to strengthen the interfacial interactions of MOF–polymer for pushing the 2008 Robeson upper bound limit. In this method, opposed to the methods above, the MOF particles were modified before incorporation into the polymer matrix, since the MMM molecular engineering of the MMM structure mainly takes place before the membrane casting process. This method benefits from the bridging technique and facilitated transport mechanisms [19], which hasten the preferential reactive gas adsorption such as CO_2_ over non-reactive gas molecules diffusion such as H_2_, N_2_, and CH_4_. 

Therefore, in this work we developed our previous concept, by fabrication of a UiO-66-NH_2_-PVA-PVAm facilitated transport mixed matrix membrane (FT-MMM), where PVA polymer chains were primarily grafted-onto the synthesized UiO-66-NH_2_ followed by embedding into the PVAm matrix. The UiO-66-NH_2_ was chosen since it provided sufficient amount of –NH_2_ groups for both providing CO_2_ fixed-carrier sites, as well as PVAm, and also its high stability in aqueous media. This method grants a robust adhesion between PVAm and the UiO-66-NH_2_ through the PVA polymer chains to the uniform dispersion of UiO-66 to form a defect-free MMM even up to 55 wt.% of MOF loading. The fabricated membranes were characterized using X-ray diffraction (XRD) analysis, Fourier-transform infrared spectroscopy (FTIR), and Raman spectroscopy measurements to evaluate the chemical interactions between the MMM components. Scanning electron microscopy (SEM) coupled with energy-dispersive X-ray spectroscopy (EDX), 3D optical profilometers were used to observe the obtained morphology and monitor the UiO-66-NH_2_ dispersion into the PVAm matrix. The gas separation performances of the MMM were measured using a gas chromatograph (TR-TC6891 N) for the separation of CO_2_/N_2_ mixture gas at atmospheric pressure and room temperature. The effect of the UiO-66-NH_2_-PVA loading amount on the gas separation performances of the MMMs was also investigated by using 0–55 wt.% of MOF–PVA amount. The overall physiochemical and gas separation properties of the fabricated MMM showed dramatic changes by enhancing MOF–polymer interface interactions using the bridging technique as an effective method to design desirable MMM defect-free morphology.

## 2. Materials and Methods

### 2.1. Materials

Commercial poly (vinyl amine) (PVAm) pellets (Lupamin-9095, Mw 340,000 g/mol) were obtained by BASF (Jakarta, Indonesia). Poly (vinyl alcohol) (PVA) (Mw: 31,000 g/mol) was provided by Sigma-Aldrich (St. Louis, MO, USA). Ethanol of 96% purity was purchased from Penta (Prague, Czech Republic). 2-Aminoterephthalic acid, zirconium chloride (ZrCl_4_), hydrochloric acid (HCl), and dimethylformamide (DMF) with 96% purity for synthesizing MOF particles were obtained from Merck (Prague, Czech Republic). All the obtained chemicals were used as received without further purification.

### 2.2. Characterization

Fourier-transform infrared spectroscopy (FTIR) measurements were conducted on a Nicolet iS50 FTIR Spectrometer (Thermo Scientific, Waltham, MA, USA) to study the physicochemical interactions between the MMM components. The membrane sample was fixed onto the attenuated total reflection (ATR) diamond crystal surface, using a deuterated triglycine sulfate detector for measurements in the range of 500–4000 cm^−1^ for all samples. X-ray powder diffraction (XRD) was performed at room temperature using a Bruker D8 Discoverer θ-θ powder diffractometer (Karlsruhe, Germany) with para-focusing Bragg–Brentano geometry with CuKαradiation (λ = 0.15418 nm, U = 40 kV, I = 40 mA). Data were scanned at an angular range of 5–80° (2θ) to determine the crystal structure of MMM by incorporating different MOF loading amounts. The morphology of the fabricated MMM was investigated by scanning electron microscopy (SEM) (Oxford instruments, Tokyo, Japan). The 3D non-contact optical surface profiler NewView 9000 (ZYGO, Prague, Czech Republic) was used for precise, quantities, non-contact surface measurement and characterization.

### 2.3. Synthesis of UiO-66-NH_2_

The UiO-66-NH_2_ MOF particles were synthesized similar to previous reports [12,18]. In brief, 6.4 g of zirconium (IV) chloride (ZrCl_4_) and 5 g of 2-aminoterephthalic acid were dissolved in 25 mL DMF separately for 1 h at room temperature. The solution was placed into a 100 mL Teflon-lined autoclave for proceeding with the solvothermal synthesis at 120 °C overnight while keeping the solution stagnant. The obtained yellow product was centrifuged for 15 min at 9000 rpm, and the precipitate was washed twice with DMF and methanol. The yellow particles were dried and activated in a vacuum oven at 150 °C for 24 h.

### 2.4. Fabrication of UiO-66-NH_2_-PVA-PVAm Mixed Matrix Membranes

Freestanding asymmetric UiO-66-NH_2_-PVA-PVAm MMM was obtained using a solution-casting method. For this purpose, the ratio of PVA: PVAm 4:1 wt.% from which 4 g of PVAm (5 wt.%) and 1 g of PVA (5 wt.%) were dissolved separately into the mixture of ethanol and water (30/70 *v*/*v*%) solvent and the solution was stirred overnight at room temperature. The desired amount of UiO-66-NH_2_ particles were added into the PVA solution and maintained for the hydrothermal process at 110 °C for 24 h. The obtained vivid yellowish-green product was filtered and washed with ethanol and water two times and dried at room temperature. The obtained UiO-66-NH_2_-PVA powder was added into the PVAm homogenous solution and left for overnight stirring at room temperature. The final solution was kept in a 20 min ultra-sonication-degassing mode for the degassing process and cast on a glassy plate using a micrometer regulating film applicator by adjusting the high on 300 μm to obtain ~40 μm average size of membrane thickness after evaporation of the solvents at vacuum oven at 80 °C for 24 h.

### 2.5. Gas Permeation Measurements

Similar to the previous report [18] the gas mixture permeation of the PVAm-PVA-UiO-66-NH_2_ membranes was conducted using a gas permeation module connected to the gas chromatograph (TR-TC6891N). The CO_2_/N_2_ mixture as the feed side passed through a humidifier and a dehumidifier at room temperature and the sweep gas to saturate with water vapor. The gas flow feed rates were fixed as equimolar fractions of a binary mixture of CO_2_ and N_2_ (50 mL/min) to the permeation cell with the diameter of 42 mm. Reproduced membranes were tested, and the average steady-state values of the measurement results were reported with deviation. The permeability was expressed as Barrer (1 Barrer = 1 × 10^−10^ cm^3^ (STP) cm/(cm^2^ s cmHg) = 7.5005 × 10^−18^ m^2^ s^−1^ Pa^−1^) as follows:

P_i_ = (Q_i_ L)/(A ∆p_i_) (1)

where Q_i_ is the volumetric flow rate of component i, L is the membrane thickness in (cm), A is the membrane area (cm^2^), and Δp_i_ is the trans-membrane pressure difference of i component (in cmHg). The membrane thickness was measured using a bench micrometer and was double-checked with SEM cross-sectional observation. The separation factor α (A/B) for mixed-gas permeation was calculated by the following equation:

α (A/B) = P_A_/P_B_ (2)

where P_A_ and P_B_ are the permeability of the gas components A and B of the permeate side.

## 3. Results and Discussions

### 3.1. Characterization of UiO-66-NH_2_ and MMMs

**Figure 1:**
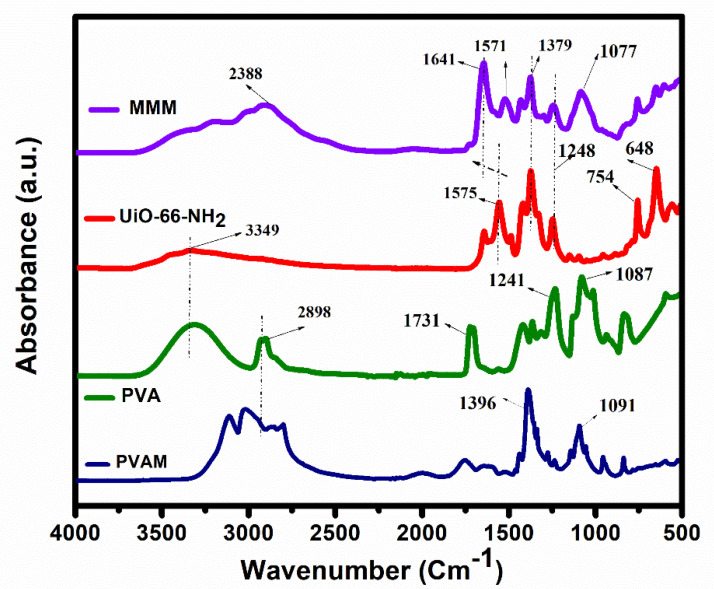
ATR–FTIR spectra of fabricated MMM and the synthesized UiO-66-NH_2_ MOF.

To study the chemical structure and the MOF–polymer interactions, ATR–FTIR and analysis were employed (Figure 1). The main characteristic peaks of synthesized UiO-66-NH_2_ were observed at 1575 cm^−1^ [ν(C=O)], 3349 cm^−1^ and 648 cm^−1^ [ν(N–H)], 648 cm^−1^ [ν(C=O)], 1383 cm^−1^ [ν(C–N)] [18]. The peaks located at 3349 cm^−1^ and 1575 cm^−1^ were assigned to the primary amines and the symmetric stretching vibration of carboxyl groups from 2-aminoterephtalic acid used for synthesis UiO-66-NH_2_. The wide band observed in the PVA sample, between 3500–3000 cm^−1^, referred to the stretching OH from intermolecular hydrogen bonds [20]. The vibration bands at 2898 cm^−1^ and 1730 cm^−1^ are assigned to the C–H from alkyl and acetate groups, respectively [21]. The large bands observed between 3200–2800 cm^−1^ and 1091 cm^−1^ were assigned to the primary and secondary existing amine groups in the PVAm sample. The observed amine groups in PVAm were connected to the hydroxyl group of PVA and afterward connected to the amines group of UiO-66-NH_2_ through the intermolecular hydrogen bonds [22]. This resulted in the linkage of UiO-66-NH_2_ to the PVAm matrix covalently, through the PVA. Created bridges via the covalent bonds between PVAm and UiO-66-NH_2_ [18] are indicated by the red-peak shift from 1575 cm^−1^ to 1641 cm^−1^ and the increased intensity of the secondary and primary amines groups (1077 cm^−1^ and 2388 cm^−1^) in the MMM samples.

**Figure 2:**
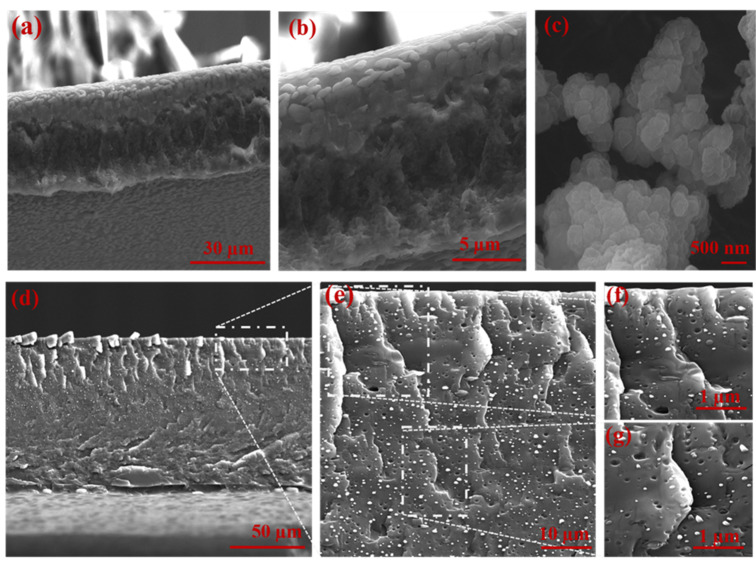
SEM images of cross-sectional view PVAm (**a**,**b**), UiO-66-NH_2_ particles (**c**), and PVAm-UiO-66-NH_2_ cross-sectional view (**d**–**g**).

**Figure 3:**
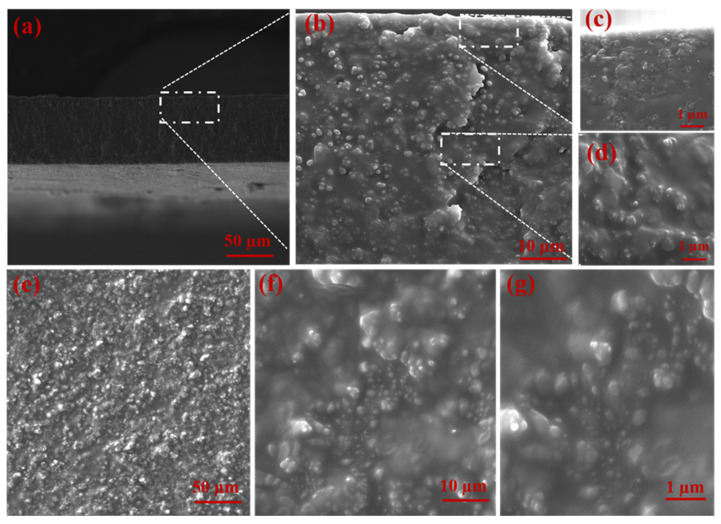
SEM images of cross-sectional view PVAm-PVA-UiO-66-NH_2_ (**a**–**d**) and the surface morphology PVAm-PVA-UiO-66-NH_2_ (**e**–**g**).

**Figure 4:**
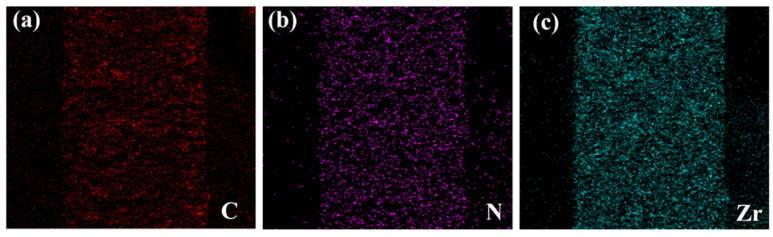
EDS of (**a**) C, (**b**) N, and (**c**) Zr elemental mapping of PVAm-PVA-UiO-66-NH_2_.

The cross-sectional view of the PVAm membrane showed a typical asymmetric structure, with a dense selective layer and porous bulk morphology (Figure 2a,b) [23,24]. The UiO-66-NH_2_ crystal structure exhibited triangular base pyramid morphology. The effect of poor and good MOF–polymer adhesion in the fabricated membranes is demonstrated in Figure 2 and Figure 3. The significant phase separation domains between MOF and the polymer matrix and the agglomerated MOF regions were observed in the PVAm-UiO-66-NH_2_ membrane (Figure 2d–g). While the homogenous MOF dispersion into the PVAm matrix was obtained using the PVA polymer chain, PVA was the connection bridge between UiO-66-NH_2_ and PVA (Figure 3). SEM analysis and further SEM-EDS mapping (Figure 4) were also applied to study the cross-sectional morphology of the MMM and MOF particle distribution. As shown in Figure 3a, the MMM of PVAm-PVA-UiO-66-NH_2_ with a thickness of ~70 µm was obtained. Unlike the PVAm-UiO-66-NH_2_ sample (Figure 2f,g), the MOF particles were dispersed improperly, forming non-selective voids and cracks into the membrane matrix.

Adversely, owing to the high interaction and strong adhesion between PVA with the MOF and the polymer matrix, appropriate dispersion of UiO-66-NH_2_ with almost no formation of sieve-in-a-cage morphology was observed (Figure 3c,d). Surface observation of the MMM in comparison with the PVAm-UiO-66-NH_2_ shown the full coverage of the non-selective domains using the PVA bridging (Figure 3e,f). The observed hillock structure (Figure 3g) was attributed to the embedded MOF particles which truly demonstrated the interfacial compatibility with PVAm through the PVA bridges polymer segments.

**Figure 5:**
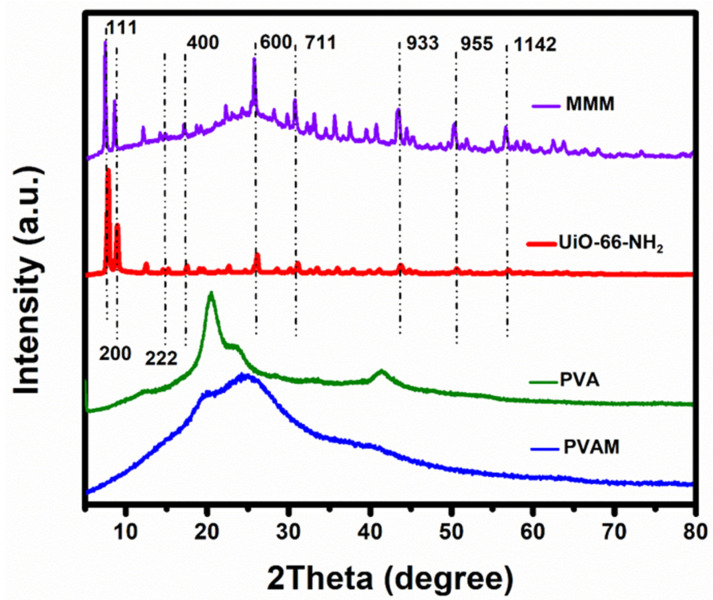
XRD spectra of fabricated MMM and the synthesized UiO-66-NH_2_ MOF.

Moreover, from Figure 3f, can be seen that the MOFs particles migrated to the surface of the membrane, which offers fast pathway channels for reactive gases with UiO-66-NH_2_ such as CO_2_. The XRD results confirmed this hypothesis since the crystallinity changed in the polymer matrix (Figure 5).

**Figure 6:**
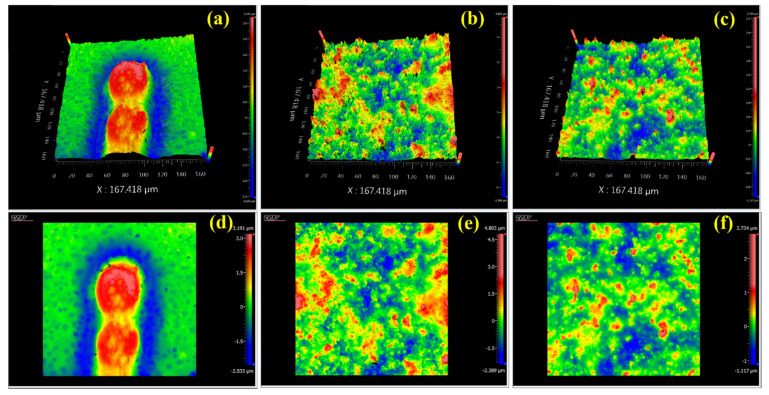
3D and 2D Optical surface profiler of PVAm (**a**,**d**), PVAm-UiO-66-NH_2_ (**b**,**e**) and PVAm-PVA-UiO-66-NH_2_ (**c**,**f**).

In other words, dispersion of MOFs particles refurbishes the polymeric membrane morphology and the crystalline lamellae domains. The MOF particle dispersion into a polymeric film was generally assumed to provide two main opposing effects, on one side increasing the free volume of the polymer, resulting in crystallinity decrement, and on the other side, effecting the polymer backbone-chain movement. Such a change in crystallinity leads to an increase in the polymer chain free volume. As shown in Figure 5, the three sharp peaks at 7.5°, 8.7°, and 26.7° in the XRD pattern for synthesized UiO-66-NH_2_ and the fabricated MMM, compared to the PVA and PVAm pristine samples, showed good agreement with the those mentioned above [25]. The optical surface profiler of samples was depicted in Figure 6. It can be seen that the PVAm membrane surface showed the freckled structure (Figure 6a), while after incorporation of MOFs the domains of UiO-66-NH_2_ were observed on the surface of the MMM. However, after using PVA as a bridges (Figure 6c) the membrane showed uniform dispersion of MOFs compared to the PVAm-UiO-66-NH_2_ membrane (Figure 6b).

### 3.2. Gas Permeation Performance of MMM

Sufficient amine functional groups provided from PVAm and UiO-66-NH_2_ enabled the characteristics of facilitated transport mixed matrix membranes (FT-MMM) in humidified conditions. The main characteristic of FT-MMM is the reversible chemical reaction of CO_2_ gas with complexing agents or carriers such as amine groups to transfer the reactive gases (e.g., CO_2_). The presence of amino groups in the membrane also leads to the homogenous dispersion of MOF particles, by strengthening the MOF–polymer interface interactions, resulting in the nearly defect-free morphology of the MMM.

**Figure 7:**
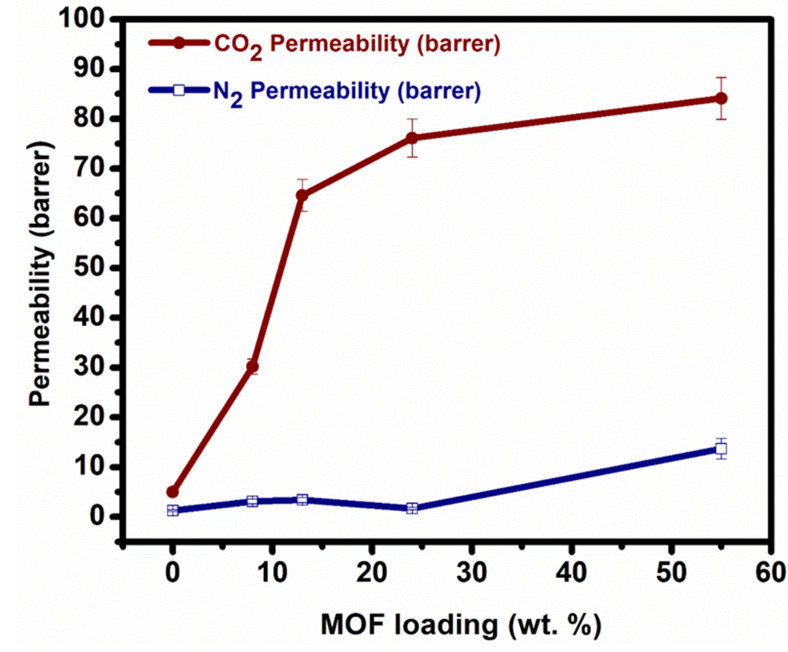
A dependence of CO_2_ and N_2_ gas permeability behavior of PVAm-PVA-UiO-66-NH_2_ MMM with different UiO-66-NH_2_ loading amounts.

On one hand, it strengthens the polymer–particle interface interactions, leading to an improvement in the uniformity and adhesion of MOF particles distribution in the polymer matrix, leading to constructing a nearly defect-free interface. On the other hand, the fixed amino group sites provide CO_2_ selective absorbability and desorbability via a reversible weak acid–base reaction resulting in high selectivity of CO_2_ per nonreactive gases. Different amounts of UiO-66-NH_2_ particles were dissolved with PVA followed by being incorporated into the PVAm matrix to conceive the effect of amino groups and MOFs loading effect on gas permeation. As shown in Figure 7, CO_2_ permeability always increased by MOFs loading increments which is due to two main reasons.

**Figure 8:**
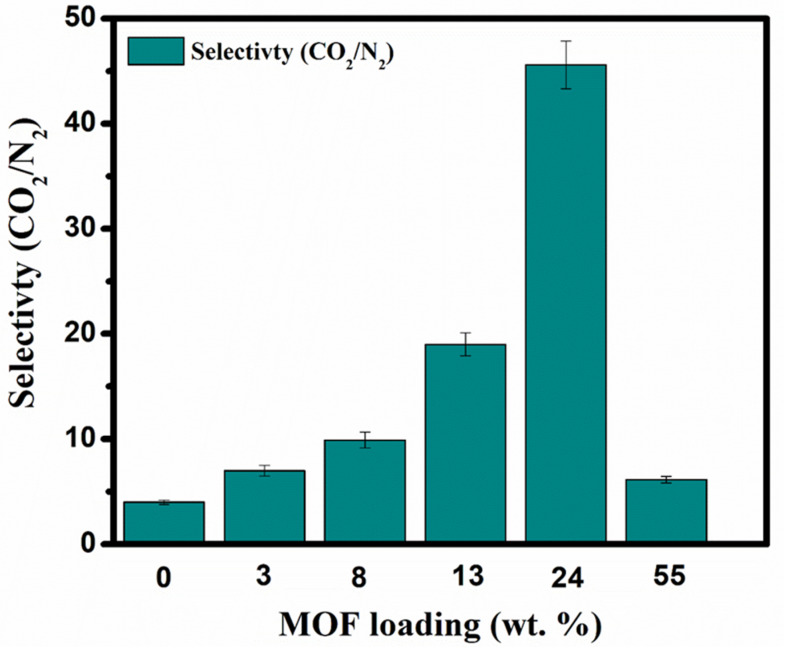
CO_2_ and N_2_ gas selectivity behavior of PVAm-PVA-UiO-66-NH_2_ MMM with different UiO-66-NH_2_ loading amounts.

**Figure 9:**
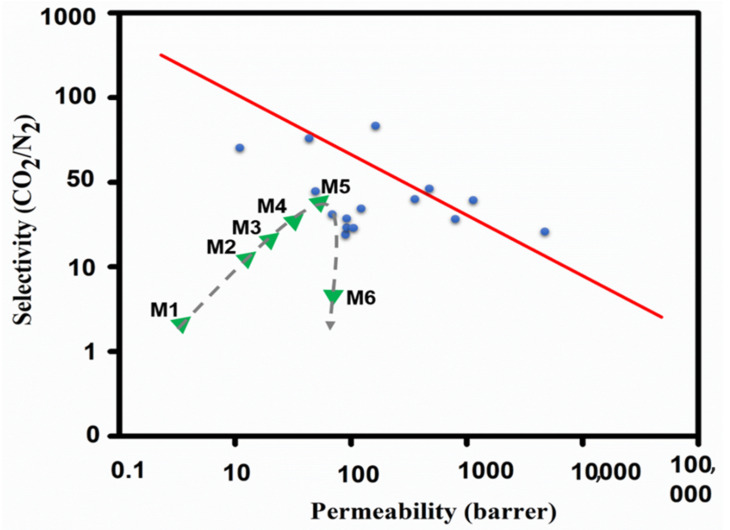
Robeson upper bound for CO_2_/N_2_ separations of M1–M6 membranes (0–55 wt.% of MOF loading). The 2008 upper bound line illustrates the tradeoff between permeability and selectivity, adapted from Refs. [9,26].

Firstly, the porous structure of UiO-66-NH_2_ provides accelerated transport channels for CO_2_ to strengthen CO_2_ permeability. Secondly, a sufficient primary amino group of MOFs reacting with CO_2_ as a CO_2_ carrier heightens the CO_2_ permeability as was discussed before. Moreover, strong polymer–MOF affinity minimalized the interfacial defects in both the selective layer and bulk of the membrane. The compact and uniform dispersion of UiO-66-NH_2_ nanoparticles on the membrane matrix offers continuous pathways for CO_2_, while it hindered the non-reactive gas, N_2_, from transferring. This led to the permeability privilege of CO_2_ over N_2_, which resulted in proper selectivity of CO_2_/N_2_ (Figure 8). The trend of the UiO-66-NH_2_ loading effect on the trade-off manner of FT-MMM is illustrated in Figure 9. The trends in Figure 9 show the relationship between MOF loading, CO_2_ permeability, and the CO_2_/N_2_ selectivity and also show the comparison of this work with the literature.

## 4. Conclusions

Our previous “bridging technique” was, in this work, successfully used for the UiO-66-NH_2_ particles bonding with PVA, and afterward incorporated into the PVAm matrix. Such MOF loading to the PVAm matrix through the PVA bridges provided the opportunity for high MOF loading up to 55 wt.% incorporation of MOF amount into the PVAM matrix, without observing any sieve-in-a-cage morphology or unwanted cracks and voids. Furthermore, the sufficient amount of –NH_2_ functional groups, provided by UiO-66-NH_2_ and PVAm, provided fixed CO_2_ carrier sites into the MMM to facilitate the preferential CO_2_ permeation through the bicarbonate reversible reaction between CO_2_ and –NH_2_ in humidified conditions. However, the non-reactive gas such as N_2_ could not easily pass through the MMM, neither by size exclusion nor solution-diffusion mechanism, due to the dense structure of the fabricated membranes as demonstrated in higher selectivity of CO_2_ over N_2_ gas molecules. The bridging technique offers a versatile and effective approach to fabricate almost defect-free MMM morphology with a high amount of MOFs loading for gas separation.

## Data Availability

Not applicable.
